# The “black cloud” on call: a field study of cognitive bias in workload perception among physicians

**DOI:** 10.1186/s12909-026-08936-y

**Published:** 2026-03-05

**Authors:** Il Woo Park, Joung Won Sung, Sangmin Lee, Min Uk Park, Bumjin Lim

**Affiliations:** 1 Department of Urology, Asan Medical Center, University of Ulsan College of Medicine, 88 Olympic-ro 43-gil, Songpa-gu, Seoul, 05505 Republic of Korea; 2 Department of Urology, Gachon University Gil Medical Center, Gachon University College of Medicine, Incheon, Republic of Korea

**Keywords:** Black cloud phenomenon, Cognitive bias, Physician perception, Workload variability

## Abstract

**Background:**

The “black cloud” phenomenon, the belief that certain physicians disproportionately experience higher workloads during on-call shifts, is a recurrent anecdote in medical communities worldwide. However, it remains unclear whether this phenomenon reflects actual differences in workload or is a subjective belief. This study aimed to verify the objective basis of the “black cloud” perception and to interpret the observed phenomenon through a theoretical framework of cognitive psychology by analyzing on-call data from physicians working in the same clinical environment.

**Methods:**

A retrospective single-center observational study was conducted on four urologists (fellows) participating in the rotating on-call schedule at a tertiary teaching hospital in Seoul from March 2024 to June 2025 (15 months). The “black cloud” perception was defined not by surveys but by behavioral indicators, specifically persistent and vocal complaints regarding workload disproportion. All shifts (*n* = 493) were standardized to 12-hour units. The primary outcome was the number of emergency calls per shift. Secondary outcomes included the frequency of early morning calls (00:00–05:30) and high-call shifts (≥ 5 calls). Statistical analyses included descriptive statistics, one-way analysis of variance (ANOVA), negative binomial regression, and sensitivity analysis.

**Results:**

A total of 493 on-call shifts were analyzed. There was no statistically significant difference in the mean number of calls per shift among the four physicians (1.25 vs. 1.12 vs. 1.21 vs. 1.22; F = 0.228, *p* = 0.877). The negative binomial regression analysis also confirmed no significant differences in the incidence rate of calls among individuals. Notably, the call frequency for Dr. Kim, who consistently expressed complaints, was numerically only slightly higher; however, this remained within the range of random variation and was not statistically significant.

**Conclusions:**

This study found no statistically significant differences in actual on-call workloads among urology specialists working in a shared environment. The discrepancy between self-perceived “black cloud” status and objectively measured workload suggests that this phenomenon may be better understood as a cognitive interpretation of experience rather than a statistically demonstrable workload imbalance.

## Introduction

Human judgment under uncertainty often relies on heuristics. While heuristics serve as adaptive cognitive strategies that facilitate efficient decision-making under constraints of time and information, they can also lead to systematic errors known as cognitive biases in specific conditions [1, 2]. For instance, the availability heuristic causes an overestimation of the frequency of vivid or memorable events, whereas confirmation bias leads individuals to selectively focus on information that supports their pre-existing beliefs [3–5]. Furthermore, the peak-end rule and negativity bias can distort the perception of an entire experience based on a few dramatic moments rather than the experience as a whole [6, 7].

These universal principles of human judgment apply to physicians, a group of highly trained experts, without exception [8]. Although physicians are expected to make objective judgments, research since Tversky and Kahneman has consistently demonstrated that expert judgment is also susceptible to bias. This phenomenon is often discussed within the framework of dual process theory; in situations involving fatigue and high stress, such as on-call shifts, physicians may rely more on intuitive, fast “System 1” thinking rather than analytical, slow “System 2” thinking, thereby increasing the susceptibility to cognitive errors [9, 10].

Such cognitive errors can form subjective beliefs that deviate from objective reality, a prime example being the widely narrated “black cloud” phenomenon in the medical community [11, 12]. Some physicians believe that an unusual number of incidents occur during their on-call shifts. This perception is found universally in hospital cultures worldwide, known as the “black cloud” in the United States, the “sh*t magnet” in the United Kingdom, and “arashi o yobu kenshui” (the storm-calling resident) in Japan. This goes beyond mere superstition, posing a significant problem that can impair individual morale, harm collegial relationships, and ultimately lead to emotional exhaustion or burnout.

This subjective belief naturally leads to a scholarly question: Is the “black cloud” an objective phenomenon that truly exists, or is it merely a psychological reality created by cognitive biases? The few empirical studies attempting to answer this question have shown conflicting results. Whereas a study in hand surgery identified a correlation between “black cloud” designation (notably among fellowship-trained attendings) and higher call burden quantified by add-on cases and transfers, an apheresis-medicine investigation detected no statistically significant associations between overnight emergent-procedure incidence and clinician- or calendar-level factors; consistent with this, a survey of surgical residents found that “black cloud” self-perception did not predict objective workload [11–13]. However, these previous studies share common limitations, such as relying on subjective self-reported data or lacking an in-depth psychological analysis of the phenomenon’s origins.

Therefore, this study aimed to systematically analyze the distribution of on-call workload among four urologists working in the same clinical setting. Specifically, we sought to verify whether a discrepancy exists between objective data and the persistent “black cloud” complaints of a specific physician. If such a discrepancy exists, we aim to interpret this phenomenon using cognitive psychological theoretical frameworks, discussing the possibility that the perceived misfortune may stem from cognitive distortions rather than objective reality.

## Materials and methods

### Study design and population

This retrospective single-center observational study was conducted at the Department of Urology of a tertiary teaching hospital in Seoul, Republic of Korea, from March 2024 to June 2025 (15 months). The study aimed to analyze the actual on-call distribution among specialists within the same work environment and to objectively evaluate the validity of the subjective perception that workload is disproportionately concentrated on specific physicians. The study population consisted of the total population of four urology specialists (fellows) who participated in the rotating on-call schedule during the specified period. Although the sample size (*N* = 4) is limited, it represents the entire cohort performing these duties. Importantly, these four specialists formed a close-knit peer group sharing a high-intensity workspace and interacting daily during their fellowship. All participants were subject to the identical on-call system within the same hospital environment, thereby perfectly controlling for systemic confounding variables unrelated to individual factors.

### Definition of ‘black cloud’ perception

Given the naturalistic origin of this study, prospective surveys to measure subjective perception were not conducted. Instead, the ‘black cloud’ perception was identified through direct, longitudinal observation of explicit behavioral indicators. Specifically, a participant was identified as perceiving themselves as a “black cloud” if they vocally and persistently expressed complaints regarding disproportionate workload during post-call conferences or daily peer interactions. Among the four participants, only Dr. Kim exhibited this behavior consistently throughout the 15-month study period. This behavioral pattern was unique to Dr. Kim and was consistently observed and corroborated by the colleagues sharing the same work environment, whereas the other three physicians did not raise such complaints.

### On-call schedule standardization

The on-call schedule at this institution distinguishes between weekdays and non-workdays (weekends and public holidays). On weekdays, each on-call specialist works a night shift from 19:00 to 07:00 the following day. On non-workdays, the 24-hour on-call duty is divided into two shifts: 07:00–19:00 and 19:00–07:00. To ensure temporal equity in our analysis, we divided each non-workday into two 12-hour “cells,” allowing all on-call duties, whether on weekdays or weekends, to be analyzed as standardized 12-hour units.

### Data exclusion and ethical considerations

To avoid confounding the analysis, exceptional cases such as emergency proxy coverage, unrecorded shift changes, and unregistered extended duties were excluded. All data were anonymized to prevent personal identification. This study received an exemption from review from the hospital’s Institutional Review Board (IRB No. 2025 − 0842).

### Data collection and variable definition

Data were automatically extracted from the hospital’s Electronic Medical Record (EMR) system and on-call schedule records. The call volume was ascertained by linking the emergency call system with ward records. Duplicate notifications and repeated entries for the same patient were cleaned during the preprocessing stage.

The primary variables were defined as follows:

- Physician: The on-call specialist (Kim, Lee, Park, Choi). To maintain anonymity, these names are pseudonyms derived from common Korean surnames. This was treated as a categorical variable, with Dr. Kim used as the reference group in the regression model.
- Date_M: The call date, converted to a year-month format (e.g., 2025-06).Seasons: A derived variable classifying each Date_M into Spring (March–May), Summer (June–August), Fall (September–November), and Winter (December–February).
- Week: A binary variable distinguishing between weekdays (Monday–Friday) and non-workdays (Saturday, Sunday, and public holidays).
- Call: The total number of emergency calls received during each 12-hour on-call cell. This served as the primary outcome variable.
- Dawn_calls: The number of calls received between 00:00 and 05:30. This was also expressed as a proportion of the total calls per shift.

All data were processed, transformed, and structured for statistical analysis using R software (version 4.5.1).

Statistical Analysis First, a descriptive analysis was performed to calculate the mean, median, standard deviation, minimum, maximum, and interquartile range (IQR) of call volumes for each physician. To assess the temporal burden of calls, the frequency and proportion of calls during the early morning hours (00:00–05:30) were also examined.

A one-way analysis of variance (ANOVA) was conducted to compare the mean number of calls among physicians, with Tukey’s Honest Significant Difference (HSD) test used for post-hoc comparisons. Given that the call volume was count data exhibiting overdispersion, a negative binomial regression was employed for the multivariate analysis. Key covariates included physician, type of workday, season, and month.

To ensure the robustness of the model, a sensitivity analysis was performed by stratifying the dataset by workday type (weekday vs. non-workday) and season.

Furthermore, to explore variability in addition to average workload, the coefficient of variation for each physician was calculated. The frequencies of high-call shifts (defined as ≥ 5 calls per shift) and no-call shifts were also analyzed. Finally, dawn calls, which may serve as an indirect indicator of nocturnal stress and fatigue, were analyzed separately.

## Results

This retrospective analysis investigated whether certain physicians consistently experience a higher on-call workload and whether the perception of being “cursed with bad calls” is statistically valid. The study included four urologists, referred to as Dr. Kim, Dr. Lee, Dr. Park, and Dr. Choi.

1. Descriptive Statistics The total number of on-call shifts (N), mean number of calls per shift, standard deviation (SD), maximum and minimum calls, and interquartile range (IQR) were calculated for each physician. The mean number of calls per physician was similar: Kim (1.25), Lee (1.12), Park (1.21), and Choi (1.22). The median was 1 for all physicians, the standard deviation ranged from 1.27 to 1.39, and the IQR was 2 for all. The highest number of calls in a single shift was 7 (Dr. Park), while the maximum for other physicians was 5–6.

As a sub-analysis, dawn calls (00:00–05:30), a common dread for night-shift physicians, were examined as a potential indicator of stress. The proportion of shifts with at least one dawn call was highest for Dr. Kim at 33.6% (46 of 137 shifts), followed by Dr. Lee at 33.3% (47 of 141 shifts), Dr. Park at 32.5% (40 of 123 shifts), and Dr. Choi at 28.3% (26 of 92 shifts).

**Fig. 1:**
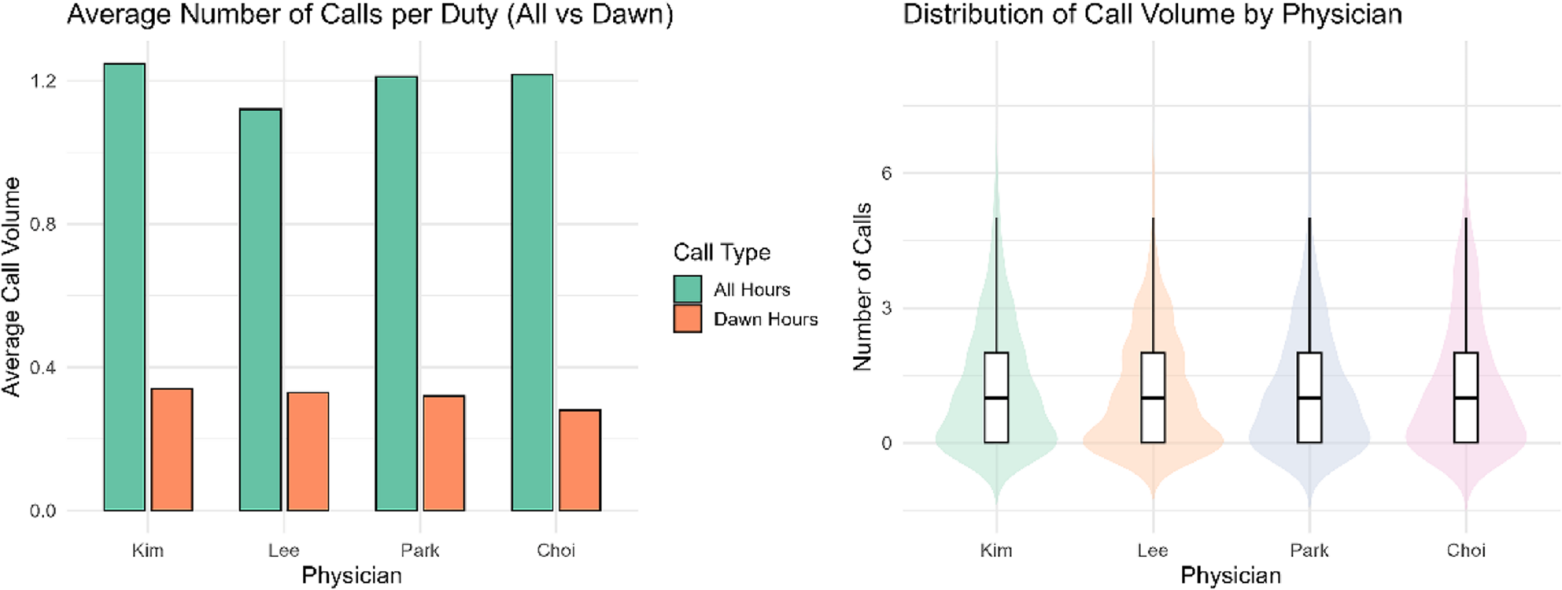
Call volumes summary by physician

Although Dr. Kim recorded the highest mean call volume and the highest proportion of dawn calls, visual inspection via box plots and violin plots did not reveal any distinct differences in the overall call distribution among the physicians (Table 1; Fig. 1).

**Table 1:** Descriptive statistics of on-call workload by physician

Physician	Total Shifts (*N*)	Mean Calls/Shift	Median	SD	Min	Max	IQR	Dawn Calls (*N*)	Mean Dawn Calls	Dawn Call Rate (%)
Kim	137	1.25	1	1.37	0	6	2	46	0.34	33.6
Lee	141	1.12	1	1.27	0	6	2	47	0.33	33.3
Park	123	1.21	1	1.39	0	7	2	40	0.32	32.5
Choi	92	1.22	1	1.38	0	5	2	26	0.28	28.3

Descriptive statistics summarising the on-call workload by physician. Metrics include total shifts (N), mean and median number of calls per shift, standard deviation (SD), minimum and maximum call counts, interquartile range (IQR), number of dawn calls (00:00–05:30), mean dawn calls per shift, and the percentage of shifts involving at least one dawn call.

2. Analysis of Variance (ANOVA) The ANOVA results showed no significant difference in the mean number of calls among the physicians (F = 0.228, *p* = 0.877). Post-hoc comparisons using Tukey’s HSD test also found no statistically significant pairwise differences between any of the physicians (all adjusted *p* > 0.85) (Table 2).

**Table 2:** ANOVA and post-hoc test results for call volume by physician

A. ANOVA Summary					
Term	df	Sum Sq	Mean Sq	F value	Pr (>F)
Physician	3	1.246	0.415	0.228	0.877
Residuals	489	890.669	1.821		

B. Tukey HSD Pairwise Comparisons				
Comparison	Mean Diff	Lower CI	Upper CI	Adj. p-value
Lee - Kim	–0.128	–0.545	0.290	0.860
Park - Kim	–0.037	–0.469	0.395	0.996
Choi - Kim	–0.031	–0.500	0.438	0.998
Park - Lee	0.091	–0.338	0.520	0.948
Choi - Lee	0.097	–0.369	0.563	0.950
Choi - Park	0.006	–0.474	0.486	1.000

A. One-way ANOVA summary comparing mean call volumes by physician

B. Tukey HSD pairwise comparisons showing mean differences, 95% confidence intervals, and adjusted p-values for each physician pair

3. Negative Binomial Regression Given that the data were count-based and exhibited overdispersion, a negative binomial regression analysis was performed. With Dr. Kim set as the reference group, the Incidence Rate Ratios (IRRs) were as follows:

- Dr. Lee: IRR = 0.91 (95% CI: 0.69–1.20, *p* = 0.498)
- Dr. Park: IRR = 0.94 (95% CI: 0.71–1.25, *p* = 0.691)
- Dr. Choi: IRR = 0.97 (95% CI: 0.72–1.32, *p* = 0.867)

**Fig. 2:**
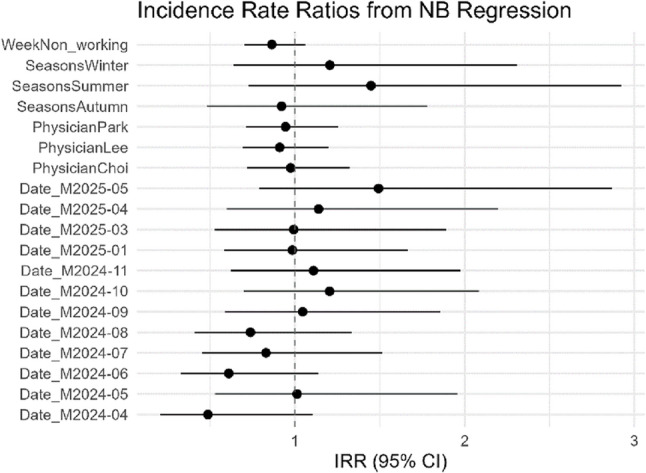
Incidence Rate Ratios (IRRs) from negative binomial regression

None of the comparisons reached statistical significance. The IRR for the workday type variable (non-workday vs. weekday) was 0.86 (95% CI: 0.70–1.06, *p* = 0.163), indicating a trend toward fewer calls on non-workdays, but this was not statistically significant. The season and month variables were also not significant (all *p* > 0.1) (Table 3; Fig. 2).

**Table 3:** Multivariable negative binomial regression for call volume

Term	IRR (95% CI)	*p*-value
Physician: Lee	0.91 (0.69–1.20)	0.498
Physician: Park	0.94 (0.71–1.25)	0.691
Physician: Choi	0.97 (0.72–1.32)	0.867
Non-working Day	0.86 (0.70–1.06)	0.163
Season: Summer	1.45 (0.73–2.92)	0.297
Season: Autumn	0.92 (0.48–1.78)	0.805
Season: Winter	1.21 (0.64–2.31)	0.569
Month: 2024-04	0.49 (0.21–1.11)	0.091
Month: 2024-05	1.01 (0.53–1.96)	0.971
Month: 2024-06	0.61 (0.33–1.14)	0.120
Month: 2024-07	0.83 (0.45–1.52)	0.540
Month: 2024-08	0.74 (0.41–1.33)	0.311
Month: 2024-09	1.05 (0.59–1.86)	0.879
Month: 2024-10	1.20 (0.70–2.08)	0.504
Month: 2024-11	1.11 (0.62–1.97)	0.724
Month: 2025-01	0.98 (0.58–1.67)	0.953
Month: 2025-03	0.99 (0.53–1.89)	0.981
Month: 2025-04	1.14 (0.60–2.20)	0.693
Month: 2025-05	1.49 (0.79–2.87)	0.224

Incidence rate ratios (IRRs), 95% confidence intervals, and *p*-values are presented for physician identity, weekend/public holiday status, season, and calendar month. Reference categories were Kim (physician), working day, and Spring

4. Sensitivity Analysis Subgroup analyses stratified by workday type (weekday vs. non-workday) and by season also revealed no statistically significant differences among physicians. In all stratified models, the IRR for each physician compared to the reference group (Dr. Kim) had a 95% confidence interval that included 1.0, indicating no significant effect (Table 4).

**Table 4:** Sensitivity analysis of negative binomial regression by subgroups

Subgroup: Weekdays		
Term	IRR (95% CI)	*p*-value
Physician: Lee	1.16 (0.85–1.59)	0.360
Physician: Park	0.96 (0.68–1.34)	0.795
Physician: Choi	0.99 (0.70–1.38)	0.935
Seasons: Summer	0.80 (0.33–2.13)	0.638
Seasons: Autumn	0.50 (0.20–1.32)	0.136
Seasons: Winter	0.80 (0.37–2.01)	0.607
Date: 2024-04	0.35 (0.07–1.27)	0.129
Date: 2024-05	0.77 (0.34–1.98)	0.559
Date: 2024-06	0.89 (0.46–1.80)	0.743
Date: 2024-07	1.00 (0.53–1.98)	0.990
Date: 2024-08	0.92 (0.46–1.86)	0.803
Date: 2024-09	1.15 (0.54–2.49)	0.709
Date: 2024-10	1.84 (0.98–3.66)	0.065
Date: 2024-11	1.45 (0.71–3.03)	0.308
Date: 2025-01	1.04 (0.57–1.87)	0.904
Date: 2025-03	0.70 (0.31–1.78)	0.413
Date: 2025-04	0.77 (0.35–1.95)	0.546
Date: 2025-05	1.00 (0.44–2.54)	0.995

Subgroup: Non-working Days		
Term	IRR (95% CI)	*p*-value
Physician: Lee	0.73 (0.44–1.21)	0.215
Physician: Park	0.93 (0.55–1.56)	0.764
Physician: Choi	1.06 (0.58–1.96)	0.855
Seasons: Summer	2.21 (0.69–7.39)	0.187
Seasons: Autumn	1.26 (0.45–3.49)	0.660
Seasons: Winter	1.50 (0.51–4.49)	0.465
Date: 2024-04	0.60 (0.18–1.98)	0.406
Date: 2024-05	1.12 (0.39–3.19)	0.833
Date: 2024-06	0.40 (0.11–1.32)	0.136
Date: 2024-07	0.71 (0.21–2.34)	0.567
Date: 2024-08	0.61 (0.20–1.77)	0.371
Date: 2024-09	1.01 (0.38–2.70)	0.977
Date: 2024-10	0.84 (0.30–2.39)	0.746
Date: 2024-11	0.89 (0.32–2.45)	0.819
Date: 2025-01	0.96 (0.35–2.57)	0.937
Date: 2025-03	1.10 (0.40–3.05)	0.854
Date: 2025-04	1.39 (0.47–4.13)	0.547
Date: 2025-05	1.91 (0.66–5.56)	0.233

Subgroup: Spring		
Term	IRR (95% CI)	*p*-value
Physician: Lee	0.89 (0.57–1.39)	0.599
Physician: Park	0.85 (0.52–1.38)	0.508
Physician: Choi	0.90 (0.52–1.53)	0.689
Week: Non_working day	0.80 (0.56–1.14)	0.210
Date: 2024-04	0.49 (0.21–1.12)	0.096
Date: 2024-05	1.01 (0.52–1.99)	0.975
Date: 2025-03	1.00 (0.52–1.92)	0.994
Date: 2025-04	1.14 (0.59–2.24)	0.703
Date: 2025-05	1.49 (0.78–2.91)	0.233

Subgroup: Season: Summer		
Term	IRR (95% CI)	*p*-value
Physician: Lee	1.00 (0.59–1.70)	0.987
Physician: Park	0.71 (0.40–1.23)	0.224
Physician: Choi	0.85 (0.44–1.62)	0.618
Week: Non_working day	0.86 (0.56–1.32)	0.488
Date: 2024-07	1.36 (0.77–2.44)	0.295
Date: 2024-08	1.21 (0.69–2.15)	0.505
Date: 2025-06	1.63 (0.88–3.05)	0.123

Subgroup: Season: Autumn		
Term	IRR (95% CI)	*p*-value
Physician: Lee	0.81 (0.43–1.51)	0.505
Physician: Park	1.21 (0.66–2.23)	0.537
Physician: Choi	1.17 (0.64–2.17)	0.609
Week: Non_working day	0.87 (0.57–1.32)	0.508
Date: 2024-10	1.21 (0.67–2.18)	0.524
Date: 2024-11	1.10 (0.60–2.02)	0.766
Date: 2024-12	1.00 (0.54–1.84)	0.995

Subgroup: Season: Winter		
Term	IRR (95% CI)	*p*-value
Physician: Lee	1.00 (0.50–1.98)	0.998
Physician: Park	1.27 (0.65–2.50)	0.483
Physician: Choi	1.03 (0.50–2.09)	0.936
Week: Non_working day	0.90 (0.56–1.46)	0.673
Date: 2025-02	1.10 (0.66–1.83)	0.710

Subgroup analyses were performed separately by weekday, non-working day, and seasonal strata. IRRs (incidence rate ratios) with 95% confidence intervals are presented in parentheses

5.Variability and Extreme Values The variability in call volume was assessed using the coefficient of variation (CV), which was 1.15 for Dr. Park, 1.13 for Dr. Lee, 1.13 for Dr. Choi, and 1.10 for Dr. Kim. The number of high-call shifts (≥ 5 calls per shift) was highest for Dr. Kim with 4 shifts, followed by Dr. Park (3 shifts), Dr. Lee (2 shifts), and Dr. Choi (2 shifts). The proportion of extreme shifts (either 0 calls or ≥ 5 calls) was 44.7% for Dr. Lee, 43.5% for Dr. Choi, and 42.3% for both Dr. Kim and Dr. Park.

## Discussion

The core of this discussion is the distinct discrepancy between reality and perception. One of the study participants, ‘Dr. Kim,’ strongly self-identified as a “black cloud” continuously from his residency through his fellowship period. This discussion aims to provide an in-depth analysis of why and how this gap occurs, using Dr. Kim’s case as a lens.

### Summary and interpretation of key findings

The analysis of objective data revealed no statistically significant differences in the on-call workload among the four urology specialists. Both the one-way ANOVA (F = 0.228, *p* = 0.877) and the multivariate negative binomial regression, which adjusted for key confounding variables like season and workday type, indicated that the individual physician was not a significant predictor of call volume.

However, Dr. Kim, one of the participants, held a strong conviction that he was a “black cloud.” This leads to the central question: “Why does a specific individual become convinced of their misfortune despite performing a statistically identical workload?” This discussion explores the possibility that the origin of this gap lies not in objective data differences but in Dr. Kim’s subjective interpretation process and cognitive characteristics.

Admittedly, he did experience the numerically highest rate of dawn calls (33.6%) and the greatest number of high-call shifts (≥ 5 calls, 4 instances) among his peers. Conversely, regarding the total number of on-call shifts, Dr. Lee performed more duties (141 shifts) than Dr. Kim (137 shifts), yet raised no complaints. However, these figures are far from statistical significance and are more accurately described as random variation; his colleagues also experienced similarly demanding shifts with comparable frequency.

The crucial point may lie in how an individual interprets unavoidable high-stress events specifically, whether they view them as a ‘special misfortune unique to me.’ From a theoretical perspective, the availability heuristic suggests that intense, negative experiences like dawn calls may be more easily retrieved from memory [3]. These accessible memories could then be selectively adopted through confirmation bias to reinforce the pre-existing belief, “See, I am a black cloud” [4, 5]. Furthermore, when considered through the lens of the peak-end rule and negativity bias, it is plausible that a few difficult on-call experiences may have overshadowed the memory of numerous uneventful ones, potentially distorting the perception of the overall workload [7, 14]. Ultimately, an event that might be perceived as just ‘an unusually tough day’ by another physician could be reinterpreted by him as ‘proof of inherent bad luck.’ This suggests that the core of the “black cloud” perception likely lies not in objective frequency, but potentially in the cognitive tendencies and interpretive differences in framing the same events into a personal narrative.

### Comparison with prior research

The academic value of our findings becomes evident when situated within the dialogue of existing research on the “black cloud” phenomenon [11–13]. Previous studies have opened the debate by showing that the “black cloud” can appear as an objective reality in certain contexts, such as the hand surgery study, while in others it is more consistent with a subjective perception without objective correlation, such as the apheresis medicine study and the surgical resident survey.

Our study makes two significant advances in this discussion. First, by using objective, EMR-based data and sophisticated statistical models in a homogeneous environment where structural variables were controlled, we demonstrate more clearly than previous research that the “black cloud” is not a statistical reality. Second, regarding the ‘reality-perception discrepancy,’ our study moves beyond mere description by applying a cognitive theoretical framework to interpret potential pathways through which such discrepancies may arise. Thus, rather than aiming to end the existing debate, this study holds academic merit by broadening the scope of the conversation from the binary question of “Does the phenomenon exist?” to the interpretive question of “How might the phenomenon be understood through the lens of cognitive psychology?”

### Observation following data feedback

A notable finding emerged during the post-study phase. When the statistical results confirming the absence of significant workload differences were shared with Dr. Kim, he exhibited an immediate receptiveness to the objective evidence. He acknowledged that his judgment had been clouded by the memory of specific high-stress shifts. Crucially, following this feedback, he ceased to identify as a “black cloud” and stopped expressing complaints about workload disproportion. While this represents a single case and should not be over-interpreted, this observation illustrates the tangible potential of objective data in correcting cognitive distortions.

### Strengths and limitations

#### Strengths

This study possesses several unique methodological strengths. First is the complete homogeneity of the comparison group. The four participants were peers of the same grade (fellows) who shared an identical work environment and patient cohort within the same department for 15 months. This effectively controlled for confounding variables such as differences in proficiency or environmental factors, thereby isolating the ‘cognitive discrepancy’ as the focal point. Second is the temporal sufficiency of the study. The 15-month duration (covering 493 shifts) was sufficiently long to encompass seasonal variations and a wide range of clinical events. This duration offsets potential biases from short-term fluctuations and enhances the reliability of the data. Third, the analytical validity was enhanced by applying a sophisticated statistical model (negative binomial regression) that accounted for the overdispersed nature of the count data.

### Limitations

Despite these strengths, this study has several inherent limitations. First, although this study included the total population of specialists performing these duties during the study period, the generalizability of our findings is limited due to the small sample size of four participants and the single-center design. Second, this study measured workload solely through the quantitative metric of ‘number of calls,’ failing to capture the ‘intensity’ or ‘complexity’ of the work. Future research should incorporate metrics that reflect the qualitative aspects of the workload. Third, this study proposes that cognitive mechanisms such as the availability heuristic and confirmation bias may have contributed to the formation of the ‘black cloud’ perception. However, our data cannot directly verify these psychological mechanisms, and other factors could also have an influence. Therefore, our discussion does not empirically verify the presence of specific cognitive biases, but rather proposes the most plausible theoretical interpretation to explain the observed discrepancy.

### Implications and suggestions

The findings of this study offer important implications for physician well-being. When a complaint about being a “black cloud” arises, it is necessary to move beyond superficial solutions. Instead, efforts should be made to understand and address the underlying psychological factors. This subjective distress is a critical signal that directly impacts physicians’ burnout and job satisfaction.

Therefore, while we cannot assert that data-driven feedback definitively prevents burnout, the case of Dr. Kim suggests the tangible potential of this approach to induce positive behavioral change. Prior to the study, Dr. Kim held a genuine conviction that he was a “black cloud.” However, immediately upon reviewing the objective evidence showing no statistical difference from his colleagues, he revised this perception, and his vocal complaints regarding workload subsequently decreased significantly.

Although this represents a single case, this observation illustrates that objective data can serve as a powerful tool to realign subjective distress with objective reality. Sharing the fact that “according to the data, everyone’s workload is statistically identical” may help physicians move away from feelings of isolation (“Why am I the only unlucky one?“) toward a more objective perspective, potentially contributing to enhanced psychological stability.

## Conclusions

This study found no statistically significant differences in actual on-call workloads among urology specialists working in a shared environment. While the present study did not directly measure cognitive processes, established frameworks such as the availability heuristic and confirmation bias offer one plausible interpretive lens through which persistent perceptions of disproportionate workload might be understood.

The findings suggest that subjective workload perceptions may not always align with objective workload metrics. Accordingly, the “black cloud” perception may be better understood as a phenomenon related to cognitive interpretation of experience rather than a statistically demonstrable workload imbalance. Data-driven feedback may provide an opportunity to compare subjective perceptions with objective information, thereby contributing to a more balanced perspective within clinical settings.

## Data Availability

The datasets used and/or analysed during the current study are available from the corresponding author on reasonable request.

## References

[1] Blumenthal-Barby JS, Krieger H. Cognitive biases and heuristics in medical decision making: a critical review using a systematic search strategy. Med Decis Mak. 2015;35(4):539–57. 10.1177/0272989X14547740 25145577

[2] Tversky A, Kahneman D. Judgment under Uncertainty: Heuristics and Biases. Science. 1974;185(4157):1124–31. 17835457 10.1126/science.185.4157.1124

[3] Tversky A, Kahneman D, Availability. A heuristic for judging frequency and probability. Cogn Psychol. 1973;5(2):207–32.

[4] Kozlov MV, Zverev V, Zvereva EL. Confirmation bias leads to overestimation of losses of woody plant foliage to insect herbivores in tropical regions. PeerJ. 2014;2:e709. 25551025 10.7717/peerj.709 PMC4277485

[5] Nickerson RS. Confirmation bias: A ubiquitous phenomenon in many guises. Rev Gen Psychol. 1998;2(2):175–220.

[6] Unkelbach C, Koch A, Alves H. Explaining Negativity Dominance without Processing Bias. Trends Cogn Sci. 2021;25(6):429–30. 33875383 10.1016/j.tics.2021.04.005

[7] Kahneman D, Fredrickson BL, Schreiber CA, Redelmeier DA. When more pain is preferred to less: Adding a better end. Psychol Sci. 1993;4(6):401–5.

[8] Bornstein BH, Emler AC. Rationality in medical decision making: a review of the literature on doctors’ decision-making biases. J Eval Clin Pract. 2001;7(2):97–107. 11489035 10.1046/j.1365-2753.2001.00284.x

[9] Graber ML, Franklin N, Gordon R. Diagnostic error in internal medicine. Arch Intern Med. 2005;165(13):1493–9. 16009864 10.1001/archinte.165.13.1493

[10] Croskerry P. A universal model of diagnostic reasoning. Acad Med. 2009;84(8):1022–8. 19638766 10.1097/ACM.0b013e3181ace703

[11] Zhao E, Tiedeken N, Wang W, Fowler J. The black cloud phenomenon in hand surgery. HAND. 2019;14(6):819–22. 29661069 10.1177/1558944718770206 PMC6900693

[12] Pham HP, Raju D, Jiang N, Williams IIILA. Black cloud vs.white cloud physicians–Myth or reality in apheresis medicine? J Clin Apheresis. 2017;32(4):235–9. 27531312 10.1002/jca.21503

[13] Asfaw ZK, Schupper AJ, Durbin J, Kellner C, Shrivastava R. Black clouds in surgery: a study of surgical resident workload and burnout. Surgeon. 2023;21(2):71–7. 36858912 10.1016/j.surge.2023.01.004

[14] Rozin P, Royzman EB. Negativity bias, negativity dominance, and contagion. Personality social Psychol Rev. 2001;5(4):296–320.

